# 21st-century capitalism: structural challenges for universal health care

**DOI:** 10.1186/s12992-019-0517-3

**Published:** 2019-11-28

**Authors:** Susan K. Sell

**Affiliations:** School of Regulation and Global Governance, The Australian National University, Acton, Australia

**Keywords:** Financialized capitalism, Global supply chains, Inequality, Intellectual property

## Abstract

The structural perspective outlined here sheds light on some of the fundamental challenges involved in achieving Universal Health Care (UHC) in this twenty-first-century era of trade and financialized capitalism. This commentary explores connections between the structure of twenty-first-century capitalism and challenges to achieving UHC, discussing three features of today’s capitalism: financialized capitalism; trade, intangibles and global value chains; and inequality (as exacerbated by the first two features). The final section discusses the various opportunities for reform to facilitate UHC—from tinkering with the status quo, to deeper regulatory reform and fundamental structural change.

## Background

This commentary presents several features of twenty-first-century capitalism, highlighting some of the challenges it poses for achieving universal health care (UHC), or access for all to appropriate health services without financial hardship [1]. The World Health Organization, the United Nations and many civil society organizations have promoted UHC as an organizing principle for national health systems [2]. Viewed in narrow terms, UHC may be restricted to “expansion of access to health care services,” whereas broader conceptions address the social determinants of health across multiple sectors and the “public health interventions needed to effectively address non-communicable diseases (NCDs)” ([3], p.1). Scholars agree that achieving UHC requires sufficient funding and an active public sector role to manage the shift from out-of-pocket expenditures to pooled health spending, including health insurance and prepaid schemes [4, 5]. McKee et al. note that, historically, health-care expansion has “tended to require a confluence of political opportunities, available financial resources (mainly from a functioning tax revenue base), and the mobilization of strong left political parties, leaders, and representatives (including trade unions)” ([6], p.S40). While some countries have made considerable progress towards achieving UHC [1, 7], the long-standing quest to achieve UHC faces new challenges in the era of twenty-first-century capitalism.

This commentary explores some of the connections between the structure of twenty-first-century capitalism and challenges to achieving UHC, focusing on three features of today’s capitalism in particular: financialized capitalism; trade, intangibles, and global value chains; and inequality (as exacerbated by the first two features). The final section discusses the various opportunities for reform to facilitate UHC—from tinkering with the status quo, to deeper regulatory reform or fundamental structural change.

## Main text

### Twenty-first-century capitalism: What’s new?

US economic and political power vis-à-vis other states in the international system has propelled the spread of financialization, trade liberalization, and global supply chains (GSCs) throughout the global economy [8]. At the macro-level, three features of twenty-first-century financialized capitalism and trade stand out. First, financialized capitalism has introduced a system of economic volatility, with repeated banking crises that threaten the real economy and households alike. Second, economic power has shifted from the mainstays of the real economy (commodity producers and traders) to the controllers of global value chains (GVCs) who own intangibles such as intellectual property and financial instruments. According to de Medeiros and Trebat ([9], p.407), “the ‘core business’ of every TNC [transnational corporation], irrespective of its particular branch, is to control and capitalize on these intangible assets” in order to maximize shareholder value and generate large rents. Third, economic concentration among TNCs fosters their oligopoly and oligopsony power vis-à-vis consumers and suppliers [8]. As a macro-regime of capital accumulation it combines “flexible labor markets with the expansion of credit … to sustain consumption in the face of stagnating real wages” ([10], p.102). This results in low wages for the many and increased economic inequality. I will discuss each in turn.

### Financialized capitalism

Neoliberal economic policies of the 1970s and 1980s ushered in an increasingly financialized global economy. The blurring of the lines between retail, or regulated commercial, and unregulated investment banking accelerated after the USA repealed the Glass–Steagall Act in 1999, thereby eliminating this 1933 firewall that had separated retail or commercial from investment banking. Led by the USA, the rise of shadow banking has introduced profound volatility into global markets. “Shadow banking” refers to a range of actors and activities, including hedge funds and money-market mutual funds that lie outside traditional banking and its public supervision [11]**.** Financial innovation has outpaced regulatory oversight [12].

Financialized capitalism is “a pattern of accumulation in which profits accrue primarily through financial channels rather than through trade and commodity production” ([13], p.174). Financial markets, motives, institutions, and elites have increasingly come to dominate the global political economy, affecting everything from production and consumption, to regulation and health [14].

New information and communication technologies (ICTs) have enabled the rapid and radical transformation of global financial markets; and virtualized trade and digitalized financial data have broadened and deepened financial markets [15]. Investment banks created new financial instruments, such as collateralized debt obligations (CDOs). Banks bought home mortgages, pooled them, and sold them as new financial products at varying levels of risk [16]. However, the market value of these instruments was obscure and non-transparent; and when the US housing market crashed, the financial sector brought the real economy down with it [8, 17]. As Storm ([18], p.303) notes, “what is most distinctive about the present era of finance […] is the shift in financial intermediation from banks and other institutions to *financial markets—*a shift from the ‘visible hand’ of relationship banking, to the axiomatic ‘invisible hand’ of supposedly anonymous, self-regulating financial markets.”

The 2007–2008 global financial crisis revealed the dubious assumptions behind allegedly “self-regulating” financial markets. In the wake of that crisis, austerity measures, cuts to welfare programs, social spending and labor market transformations have had negative effects on domestic health equity and health outcomes [16]. Greece and other countries have been forced to adopt austerity measures and reduce public spending sharply; health budgets have been particularly hard hit [19]. As McKee et al. point out, debt crises resulting from costly bailouts of the financial sector “have strengthened the arguments of those promoting the case that existing welfare systems are unaffordable” ([6], p.S43).

Financialization primarily affects the financing and provisioning of UHC. One major barrier to expanding access to health care globally is its considerable costs [2]. UHC has been funded primarily by tax revenues that governments may use to implement redistributive policies in support of UHC. As Savedoff et al. point out, “the predominance of pooled spending is a necessary condition (but not sufficient) for achieving universal health coverage” ([4], p.924). Risk pooling “reallocates funds from healthy to sick individuals” ([4], p.926). Pooled health financing has two positive effects on health care. First, it “contributes to higher health spending by increasing the effective demand for health care services”; second, it can “improve health at lower costs” by emphasizing cost-effective prevention of poor health and negotiating fees and prices ([4], p.929). The World Health Organization (WHO) has noted the promise of financing health through high-risk pools for achieving UHC [5]—which require adequate domestic tax revenue. As Reeves et al. have found, “tax revenue was a major statistical determinant of progress towards universal health coverage” ([20], p.274).

Financialized capitalism has posed notable challenges to domestic tax bases. Capital mobility has facilitated tax evasion and the opportunities for shifting revenue to tax havens or low-tax locations, thereby reducing the tax base available for programs like UHC. In countries such as the USA and the UK that pioneered and promoted financialized capitalism, policymakers have cut taxes for the wealthiest and have allowed their most profitable companies to evade taxes. Tax revenues that *should* be generated in home as well as host countries are not materializing, due to widespread tax evasion. An estimated “40% of multinational profits are shifted to low-tax countries each year” ([21], p.34). It has been calculated that 500 of the largest US firms are holding “2.1 trillion offshore, avoiding an estimated $520 billion tax liability” ([22], p.228). The increased prevalence of tax avoidance by TNCs means that public budgets are further stretched to provide social goods and services. “As global GDP rose over the past decade, global tax revenues, as a portion of global GDP, fell from 15.7% in 2001 to 13.6% in 2010, corresponding to a loss of US$1.4 trillion in revenue, enough to finance UHC at current estimates” ([20], p.279).

Financialized capitalism also is predicated on the concept of shareholder value: those who invest in shares in firms expect that the firm’s profits will increase the rate of return on their investment. Shareholder value is assessed quarterly, which biases decision making toward short-term gains. As Durand and Milberg ([21], p.34) point out, “US firms in particular have had high levels of profit and cash flow in the past 15 to 20 years associated with a disproportionately large payout to shareholders in the form of dividends and share buybacks and sluggish investment.” Pressures to deliver shareholder value mean that these firms have incentives to “reduce their physical and labor footprint in order to maximize returns on a much smaller set of physical assets” ([23], p.209). For instance, in 2014, 14 of the 15 US-based firms with the largest cash holdings were firms that “rely on intellectual property rights for their profitability” ([23], p.204). The top ten US firms by cash holdings in 2014 were all either Big Tech (with Apple in the lead) or Big Pharma [23]. Monopoly power can promote economic stagnation to the extent that firms relatively immune to competitive pressure are “less compelled to invest” in the real economy ([21], p.34).

These trends have additional tax consequences. Reduced labor and physical footprints mean a reduced tax base from immobile assets such as physical property, and income tax revenue is reduced on depressed wages. Governments keen on funding UHC in these circumstances have sometimes turned toward indirect taxation, such as consumption taxes. However, Reeves et al. found that regressive consumption taxes were adversely associated with child survival; by contrast, taxation on income, capital gains, and profits had a positive effect on health spending: “each $100 rise in taxation was correlated with an $16.70 increase in health expenditure from income, profits and capital gains” ([20], p., 275). These findings strongly support government efforts to shift from regressive consumption taxes to a focus on profits and capital gains. However, governments seeking to attract foreign direct investment often fear that such tax policies might deter investors. Thus political and economic pressures may serve as disincentives for adopting optimal policies for UHC.

At the micro-level, financialized capitalism looms large in the daily experiences of more and more households—what van der Zwan calls the “financialization of the every day,” which includes “a shift towards financial markets for the provision of people’s basic needs” ([10], p., 111). The culture of individual responsibility for outcomes “places the merits of success and the burdens of failure on isolated individuals, and suggests that the resolution of every social problem requires further individualization and financialization of social provision and intercourse” ([24], p.697). These trends have effectively redefined “the citizen as a consumer of a bundle of services rather than a member of society with collective obligations and rights” ([19], p.354).

The discourse of “self-regulation” has been powerful, accompanied by “the legitimating narratives about democratization of finance and marketization of risk” ([17], p.101). Consumer credit, mortgage loans, retail investment banking, microfinance initiatives, and additional initiatives to promote financial inclusion are all aspects of this terrain. The “democratization of finance” refers to the increased availability of credit for consumers and deeper integration of the middle and working classes into the financial system. Consumers are bound to financial markets through credit-card debt, home mortgages, student loans, and the increased individual responsibility for providing for their own retirement. This has made ordinary citizens more dependent on the success of the financial sector overall, and debt-financed consumer demand has been driving economic growth in the USA and the UK [19].

Households at the middle and lower end of the income scale have increasingly financed their expenses by assuming mortgage and credit-card debt: “between 2003 and 2007, US consumer debt more than doubled” ([25], p.17). As De Vogli and Owusu ([25], p., 17) point out, “while promoting consumers’ indebtedness, times of high economic inequalities provide the wealthy with large amounts of surplus capital to invest in short-term gains and highly speculative financial assets.” According to Lavinas ([26], p., 504), “debt appears to be essential to survive or to thrive, to escape poverty as well as enjoy economic security. In parallel, redistributive conflicts will continue to be masked by growing access to credit and loans.”

In recent years “financial inclusion” has become a prominent approach to pro-poor development policies. Poverty has been re-imagined as the lack of access to a bank; “‘public’ has become increasingly private and has been taken over by finance” ([26], pp.508, 503). Microcredits, microfinance, and conditional cash transfers have expanded throughout the global economy, at the expense of “a risk-sharing system based on progressive taxation and comprehensive welfare systems” ([26], p.512). “Monetized debt relationships are becoming increasingly prominent in people’s daily lives, via the growth of consumer debt, microcredit initiatives, savings clubs and debt collection agencies” ([27], p.2). As Mader points out, “inclusive financial markets by no means automatically offer poor people a ‘fair’ deal; they generally offer lower-quality services at higher prices” ([28], p.464). Households and individuals experience the financialization of the everyday through price pressure, low incomes, debt obligations, and job insecurity.

The financialization of health is evident in the issuance of Ebola Bonds to fund pandemic risk, and the dramatic spike in crowdfunding for health care. Both examples highlight the lack of more sustainable alternatives of public provision. In a financial world of mobile capital, “trading can easily be extended and there is a perpetual search for the next ‘new, new thing’” ([15], p., 47). It seems we are witnessing the financialization of everything, with banks issuing “cat bonds” for catastrophic climate-related disasters, or the World Bank’s Pandemic Emergency Funding Facility that allows investors to purchase “Ebola bonds” [27]. The World Bank raised US $320 million for pandemic bonds; its president Jim Young Kim explained that the initiative was a way of “leveraging our capital market expertise to serve the world’s poorest’” [29]. The rise of so-called Social Impact Bonds supports the goal of narrowing public services expenditure gaps by making ethical profits [26]. However, the Ebola Bonds have failed to pay out, and critics have noted the opportunity costs of relying on such schemes. As Bodo Ellmers of the European Network on Debt and Development stated, “‘the financialization of risks is a new avenue for the privatization of profits and the socialization of losses; it would be better if donors funded the necessary assistance directly’” [29]. Tim Jones of the Jubilee Debt Campaign concluded that under these schemes “‘the public sector is always likely to lose’” [29].

Crowdfunding through platforms such as GoFundMe and KickStarter raised “US $16.2 billion in 2014, up 167% from US $6.1 billion the previous year” ([30], p.48). GoFundMe, one of the largest crowdfunding forums, “raised US $147 million for medically-related projects in 2014, up from US $6 million in 2012” ([30], p.50). The most popular segment of these platforms concerns health-related expenses for patients unable to afford medical services or products such as cataract surgery, chemotherapy, and household accessibility adaptations, (26% of all donations) [30].

While some may see this trend as positive because it increases participation in healthcare, it also points to massive structural failure that denies individuals viable alternatives. In the USA, crowdfunding has been found to avert between 114 and 136 bankruptcies per quarter (medical expenses were the leading cause of US bankruptcies in 2014); “a higher proportion of these US medical expense campaigns are hosted by patients located in states without the Affordable Care Act Medicaid expansion” ([30], p.51). Clearly, the surge in crowdfunding is symptomatic of “a failure of publicly-funded health systems” ([31], p.240).

### Twenty-first-century trade, intangibles and global value chains

Twenty-First-century trade and financialized capitalism have engendered profound structural change in the global economy. As Johns and Wellhausen ([32], p.33) point out, as of 2016 “MNC-coordinated supply chains account[ed] for 80 percent of global trade.” Power in GSCs and vertical production networks accrues to those who control “intangible assets related to innovation, finance, and marketing” ([9], p.401). While the mechanics of their rise and contemporary dominance are not identical, the big global players in IP and finance share similar political goals.

What has changed dramatically under twenty-first-century trade and financialized capitalism is the share of revenue going to those who control intangible assets. With the outsized role accorded to intangibles such as financial products and intellectual property [1], twenty-first-century trade entails what Baldwin ([33], p., 9) has characterized as the “trade–investment–services–and intellectual property nexus.”

This shift towards intangibles, as opposed to commodity production and trade in goods, has shifted economic and political power towards the owners of intangibles, with negative effects on health. In particular, it has undermined the political power of labor and trade unions, enhanced the power of capital, and introduced greater uncertainty in the commitment to public provision of goods and services. Government are criticized for being both too big and too inefficient [7].

Twenty-First-century trade exhibits new features made possible by technological change and capital mobility. The rise of GSCs has resulted in a “spatial sorting of skill-intensive stages to high-wage nations and labor-intensive stages in low-wage nations” ([34], pp.13–14), fundamentally altering the political economy of trade. Trade liberalization used to be all about reciprocal market access—“‘I’ll open my markets if you open yours’”—but, with the rise of GSCs, many emerging economies have *unilaterally* adopted liberalizing reforms to attract foreign direct investment, factories, and jobs ([34], p.9). Countries seeking to become factories for GSCs have adopted pro-supply chain policies such as assurances for intellectual property protection and liberalized capital movements, or have yoked themselves to bilateral investment treaties and regional trade agreements that fortify such assurances [33]. Services trade partisans could now claim that many domestic regulations functioned as “non-tariff barriers” to banking, data processing, and other sectors of services trade ([35], p.48). This can reduce policy space for adopting pro-health domestic regulation.

The global value chain can be conceived as moving along the following sequence: R&D, design, logistics/purchase, production, logistics/distribution, marketing and services. According to “smile curve economics,” in the 1970s, the share of revenue going to manufacture, or commodity production, was not so different from the share that went to advertising, logistics, and services [34]. By contrast, in the twenty-first-century, the bulk of the profits goes to controllers of intangibles such as R&D, intellectual property (e.g., copyright, patents, trademarks) and services. “The pre- and post-fabrication stages consist primarily of services rather than goods” ([34], p.19).

Owners of intangibles have gained monopoly and oligopoly power through the dramatically strengthened protection and enforcement of intellectual property that these owners successfully lobbied for in the 1970s and 1980s [36]. The 1994 Agreement on Trade-Related Aspects of Intellectual Property Rights (TRIPs) in the World Trade Organization ushered in a new era of “intellectual monopoly capitalism” [37], facilitating the rise of today’s hierarchical division of labor that “generates wild competition at the lower value-added stages of production, where low wages and profit margins prevail for workers and suppliers operating out of export processing zones in developing countries” ([9], p., 401). There has been a *relative* de-industrialization of the Global North and a re-industrialization of the Global South. Rather than having to *build* global supply chains, countries in the Global South have *joined* them [34].

What does this mean for UHC? As McKee et al. note, “many low- and middle-income countries’ economic models have … relied heavily on extensive foreign investment and integration in global markets, which has constrained their ability to raise taxes and public revenue, a critical precondition for establishing a viable UHC” ([6], p.S43).

What we talk about in discussing “trade” these days is really the control of intangibles such as financial services and intellectual property. Trade agreements now have little to do with trade, and everything to do with deep economic integration and domestic regulation. As Rodrik ([38], pp., 75–76) points out, “trade agreements are shaped largely by rent-seeking, self-interested behavior on the export side”: the main beneficiaries are those who control global value chains (GVCs), including international banks, Big Pharma, Big Food, Big Tech, and TNCs. According to Durand and Milberg ([21], pp., 21–22), “lead firms engaged in GVC trade are interested in stricter IPRs in trade agreements to contain the risk of IP appropriation resulting from the international fragmentation of production.” Today, “profitability is a function of a firm’s ability to extract monopoly rents from complex value chains using their control over IPRs” ([23], p.197).

Big Pharma routinely blocks pro-health initiatives aimed at promoting the use of TRIPs flexibilities to make essential medicines affordable, because these would threaten their profits and reduce shareholder value [39]. These private actors also press for provisions in plurilateral and bilateral trade and investment treaties that curtail policy space for pro-health initiatives [40]. Investor-State Dispute Settlement [ISDS] agreements produce regulatory chill: policymakers may choose not to adopt pro-health or pro-environment regulations that might expose them to costly ISDS litigation [41].

Commercial returns mean “a mismatch between health research priorities and the burden of disease outside the industrialized world” ([42], p.S16). Patent protection increases the prices of drugs and reduces access to medicines and vaccines. Strategic behavior aimed at blocking generic competition contributes to rising drug prices: “companies create serial barriers to hold off competition” ([43], p.7). “When more than 70% of best-selling drugs had their protection extended, it is clearly the go-to approach for profitability” ([43], pp.49–50). Trade agreement provisions that extend patent life and impose clinical test data exclusivity all serve to suppress price competition for drugs and medical devices, thereby reducing access to them, and support high rents for Pharma. According to Feldman, “our incentive structure is badly misaligned with societal goals” ([43], p.54).

The economic and political power of IP-rich global firms has enabled them to shape trade and investment agreements to further entrench their power and increase their profits. According to Missoni, “trade and investment treaties limit the policy space for public regulatory interventions to protect public health” ([42], p.S15). Trade and investment agreements routinely contain intellectual property provisions that extend protection beyond the TRIPs obligations of the World Trade Organization. Intellectual property has come to be treated as an “investment asset” in Investor-State Dispute Settlement (ISDS) agreements under which private firms can sue states for regulations that they claim reduce the “expected value” of their initial investment. The Philip Morris tobacco cases against Uruguay and Australia over plain packaging (thus reduced value of the trademark) and the Eli Lilly case against Canada over its pro-health patent policies are three recent examples of intellectual property-based challenges to public health regulations [44]. Even though Philip Morris and Eli Lilly lost these ISDS challenges, we can expect to see more IP-based ISDS challenges in the future [44].

### Inequality

Many scholars have noted sharply increased income inequality both across and within countries. One of the most profound changes in the global economy in the past 40 years has been the re-industrialization of the Global South and the relative economic convergence between North and South, with China leading the way—but stark differences between “headquarter countries” and “factory countries” remain ([34], pp.7–8). Even within the Global South, trends are uneven, with some countries experiencing premature de-industrialization and falling wage shares [45]. Inequalities within countries persist and sharpen.

Income inequality reduces the social solidarity essential to UHC. “UHC coverage implies a sense of solidarity and interconnectedness within a society as members agree to pool resources to guarantee at least an acceptable level of response to those in need” ([7], p.S36). Inequality is a barrier to social comity, because “wealthy elites in fast-growing yet highly unequal countries are able to opt for high-priced private care, creating a system where they see little benefit in having their tax funds invested” ([6], p.S43).

Structural changes in the global economy include declining labor power as footloose global corporations seek low-wage production abroad. Producers’ profit margins drop, given the competition to keep buyers on board with very low prices; this exerts downward pressure on returns to producers and their employees’ wages. Twenty-First-century trade and financialized capitalism reinforce short-termism due to the quarterly shareholder value pressure, as well as further eroding the already weak link between labor productivity and wage growth. The shift toward “flexible” employment and downward pressure on wages has sharply reduced the political and economic power of labor. Profitability requires firms to reduce their labor and physical footprints [22, 23].

Increasing income inequality has facilitated new patterns of consumption, with negative effects on health and greater pressure on health-care systems. The spike in such non-communicable diseases (NCDs) as diabetes, heart disease, and obesity places more stress on health-care systems. Fast food and ultra-processed food have contributed to the global rise in NCDs. “Income and its distribution, income inequality, economic insecurity, and unhealthy lifestyles link trade policy to social determinants of health” ([42], p. S15). As Pirie points out, “the growth of income inequality has had a far more direct role in shaping the development of the fast food industry” ([46], p.846). Two mutually reinforcing dynamics propel the global spread of the fast-food industry: it depends upon an ample *supply* of low-wage flexible labor, and these poorly paid workers create additional *demand* for cheap, filling food [46]. The association between “increases in obesity, reductions in time spent in food preparation and a shift toward consumption of processed foodstuffs” is well-documented in both developed and developing countries ([47], p.112).

Trade liberalization has accelerated the adoption of “unhealthy ‘Western lifestyles’ and a worldwide increase in chronic diseases, with a heavier burden in poor countries” ([42], p.S15). Economic concentration among GVC controllers has meant that, “food production and processing is dominated by a small number of large, multinational agribusiness corporations that have global reach, a massive investment in increasing productivity …and an economic and scientific orientation towards feeding mass populations rather than local communities” ([47], p.122). The dominance of supermarket chains and fast food outlets has enhanced “access to obesogenic and highly profitable ‘ultra-processed’ foodstuffs” ([47], p.113). These developments have had the effect of “transforming the means of satisfying a physical need for food into a market activity populated by food consumers” ([47], p.123).

Biomedical public-health discourses and industry narratives around individual responsibility and choice-centric conceptions about consumption obscure these structural dimensions. As Pirie notes, “medicalization and biomedicalization obscure the role of capitalism in generating these problems [eating disorders] and encourage a focus on individual dysfunction” ([46], p.839). These approaches “reduce the pressure on the state to intervene in the market” ([46], p.841).

### Spectrum of reform opportunities

Twenty-First-century capitalism seems to be driving us further away from the goal of Universal Health Coverage, shifting obligations from the public provision of basic services to private households and individuals to borrow money to “invest” in their health and wellbeing ([26], p.509). Nolke et al. ([48], p., 216) hold: “for financialization to be sustained, it invariably needs to incorporate new areas in terms of other economic sectors, the public sector, social security systems, the housing markets or other spheres of social reproduction—and reorganize them according to the rationality of financial markets.”

As this brief review has highlighted, twenty-first-century trade and financialized capitalism have not served the masses particularly well. A very small percentage of people have benefited enormously, while sharpening income inequality has left the vast majority behind. This is not to detract from the notable and spectacular gains in incomes in China and India, which have lifted many millions of people out of grinding poverty. However, even in those countries, the gaps between those at the top and those at the bottom are widening sharply and rapidly. Twenty-First-century trade and financialized capitalism have played a prominent role in exacerbating income inequality. Given the recent spread of right-wing populism and the increasing incidence of “deaths of despair,” [49] we need to ask whether “the inequality of contemporary capitalism is reaching levels that may threaten the social conditions required for the existence of democratic societies” ([37], p.1427).

What reform opportunities are available to put the vast majority of the people, and our planet, on firmer footing? Reform options lie on a spectrum. At one end lies business as usual—the status quo. This may involve minor tinkering, akin to re-arranging deck chairs on the Titanic. Some have touted the promise of corporate social responsibility initiatives and private–public partnerships. However, the rise of “corporate social responsibility” initiatives and the plethora of public–private partnerships have served to strengthen the hand of capital in multilateral health policy-making. Controversies over the Gates Foundation’s outsized role in the WHO are just one example of the tensions that can arise in an environment of shrunken public budgets and dependence on private-sector donors for multilateral governance. We find another example of a status-quo orientation in European discussions of some procedural reforms to the Investor-State Dispute Settlement system, including a multilateral investment court and various ways of selecting investment arbitrators [41]. However, these reforms will do little to fundamentally alter the dynamics of the system. Further post-crisis reforms, including new capital requirements for banks and regulations on Over-the-Counter derivatives, do not challenge the underlying conditions that make financialization possible [8, 48]. Tepid reform proposals assume that “a globalized and liberal financial system can work well, if the incentives are correct, regulation well-constructed and sufficient information available and transparent” ([48], p.214).

Somewhere in the middle of the spectrum lies more thoroughgoing regulatory change. Two of the problems affecting efforts to expand UHC have been financial market volatility and reduced tax revenue. Financial stability and adequate tax revenue are necessary (if not sufficient) conditions for achieving UHC [4, 20]. As put by Gerald Epstein [50], meaningful change would include converting “roaring banking” back into “boring banking.” The era of boring banking (1940s–1970s) restricted the risks that banks were permitted to take, and featured public missions to provide housing finance and long-term credit, and interest-rate ceilings and restrictions on competition that guaranteed a moderate rate of return and stability in the sector [50]. As regards the poor behavior of bankers and traders shaped by incentives that have included huge compensation packages for very risky activity, reforms should focus on re-regulating the financial sector [15]. The compelling analysis conducted by Farhi and Tirole [11] demonstrates that “ring fencing” basic retail and commercial banking systems from investment banking risks would prevent the contagion of collapse that spread from the shadow banking to the regulated banking sector, in the market crash of 1929 and the Global Financial Crisis of 2007–2008. Bringing back the Glass–Steagall firewall between commercial and investment banking would mark a step in this direction. Beyond ring fencing, Epstein [50] recommends more far-reaching changes in the regulation of the banking sector. These include reducing the size of “too big to fail” banks, imposing taxes on financial transactions to increase public revenue, and implementing asset-based reserve requirements.

Beyond banking, reforms of the pharmaceutical and food sectors could have a positive effect on UHC. Curbing the abuses of monopoly power in the pharmaceutical sector through pricing transparency and price reductions for medicines would help to increase access to essential medicines. Alternative financing mechanisms for drug development, such as prize funds, and the creation of patent pools could be developed further. Reforms in the food industry might include higher wages for farm and restaurant workers, a commitment to worker-owned food businesses, and the restriction of harmful business practices that promote unhealthy consumption [47].

A further option would be social mobilization to regain control over assets vital to health, construed more broadly. The case of the privatization of water is instructive here [42]. Beginning in the early 1990s, the World Bank’s International Finance Corporation began to push a commodified conception of water: instead of being considered as a human right, water became just another commodity for purchase [51]. Privatization of water resources and MNC control over formerly publicly provided water have provoked violent clashes and international legal disputes [52]. Price increases in privately-held water resources have led to 180 cities and communities in 35 countries terminating private water agreements and returning control to the public sector [51]. Civil society has been mobilizing against some of these structural trends.

However, many argue that deeper structural reform is necessary. At the more radical end of the spectrum would be reforms going beyond sectoral or grassroots reform, to address underlying structural conditions. If the problems are macro-structural, then the solutions must be, too. Critics have argued that the current focus on financing a limited UHC is too narrow a vision. According to Bloom et al.:

The UHC scope … avoids the question of ensuring universal access to many public health interventions that could lead to healthier lives—including health education campaigns, in-home piped water supplies, regulation of excessive sugar and salt in the food supply, tobacco control, road traffic safety, construction of walkable cities, high-quality primary and secondary education, and equitable distribution of wealth ([2], p.5).

We might envisage a set of reforms that would institute progressive taxation, re-commit to the public provision of basic necessities, including UHC, and to the de-commodification of goods and services such as water, education, and healthcare. Dean Baker [53] advocates full employment; addressing inequality by curbing high-end rents in the financial sector; adopting new approaches to innovation that eliminate the monopoly power of IP; sharply reducing executive compensation; and remembering that today’s policies are the result of government policies and political *choices.* This suggests that it is possible to make different choices.

In thinking about reform, it is important to ask: “what is the financial system even for?” ([54], p.359) Is it for the few or for the many? Can it promote human well-being? In order to prioritize human well-being one must first re-imagine what we “count,” and why. A recent international movement is working to develop new metrics that go beyond traditional measures such as GDP. The United Nations’ annual Human Development Reports have been important contributions in this endeavor. The OECD [55] has developed a “Better Life Index” that addresses issues such as trust, insecurity, inequality and sustainability. Alluding to the “deaths of despair,” Joseph Stiglitz [56] has noted, “had the US, for example, focused more on health, rather than just GDP, the decline in life expectancy among those without a college education, and especially among those in America’s deindustrialized regions, would have been apparent years ago.” When viewed through a prism of security and equality, pension “reforms” that increase individual risk, labor “flexibility” that results in insecure employment and lower wages, and  “austerity” that curtails public spending on welfare and punishes citizens to make banks whole—all these look far less desirable than when seen through the narrow lens of GDP [56]. As James K. Galbraith [57] has argued:

The way forward is a program for growth and justice built on the needs of the working population and the middle class.… The economic commitment … must be to full employment here [USA], to egalitarian growth in Europe and Japan, and to a worldwide development strategy favoring civil infrastructure and the poor. Public capital investment, stronger unions and a high minimum wage should frame the domestic agenda. Overseas, crackdowns on tax havens and the arms trade, a stabilizing financial system and an end to the debt peonage of poor countries should be among the priorities of a new structure.

Directly addressing inequality would help to reduce policies that exacerbate it, as “wealthy populations are, on average, more likely than the general public to oppose policies concerning taxation, social welfare, and economic regulation” ([34], p.17). As De Vogli and Owusu ([34], p.26) put it, “to reduce inequality at the root of financial instability and health problems, it is necessary to have some ‘austerity for the rich and big finance’.” Redistributive policies will be vital to expanding UHC and to addressing the broader social determinants of health.

## Conclusion

The structural perspective outlined here sheds light on some of the fundamental challenges for achieving Universal Health Care. The concepts underlying twenty-first-century trade and financialized capitalism help to explain the key players, the concentration of economic and political power, the control of intangibles and their skyrocketing value—all of which will have to be addressed if the goal of UHC is ever to be realized.

## Data Availability

N/A.
